# Supplementary material for: Epidemiology of syphilis infections among pregnant women in Tanzania: Analysis of the 2020 national representative sentinel surveillance
Source: PLoS One. 2023 Aug 31;18(8):e0285069. doi: 10.1371/journal.pone.0285069 (PMC10470872; doi:10.1371/journal.pone.0285069)
Supplement: S1 Checklist — (DOCX) [file pone.0285069.s001.docx]

STROBE Statement—checklist of items that should be included in reports of observational studies

|  | Item No. | Recommendation | Page  No. | Relevant text from manuscript |
| --- | --- | --- | --- | --- |
| **Title and abstract** | 1 | (*a*) Indicate the study’s design with a commonly used term in the title or the abstract | 1, 2 | As indicated in the title and abstract this was the analysis of the 2020 national representative sentinel surveillance. |
|  |  | (*b*) Provide in the abstract an informative and balanced summary of what was done and what was found | 2 | Indicated in the abstract. |
| Introduction | | | |  |
| Background/rationale | 2 | Explain the scientific background and rationale for the investigation being reported | 3-4 | As indicated in the introduction |
| Objectives | 3 | State specific objectives, including any prespecified hypotheses | 4 | Line 79-80 |
| Methods | | | |  |
| Study design | 4 | Present key elements of study design early in the paper | 4 | This has been reported under the section “Surveillance duration and site selection” |
| Setting | 5 | Describe the setting, locations, and relevant dates, including periods of recruitment, exposure, follow-up, and data collection | 4,5,6 | These have been reported under different sections including; surveillance duration and site selection, Training of research personnel, Data collection, Blood testing procedures. |
| Participants | 6 | (*a*) *Cohort study*—Give the eligibility criteria, and the sources and methods of selection of participants. Describe methods of follow-up  *Case-control study*—Give the eligibility criteria, and the sources and methods of case ascertainment and control selection. Give the rationale for the choice of cases and controls  *Cross-sectional study*—Give the eligibility criteria, and the sources and methods of selection of participants | NA  NA  4 | This has been elaborated under the section; Survey population |
|  |  | (*b*) *Cohort study*—For matched studies, give matching criteria and number of exposed and unexposed  *Case-control study*—For matched studies, give matching criteria and the number of controls per case | NA | NA |
| Variables | 7 | Clearly define all outcomes, exposures, predictors, potential confounders, and effect modifiers. Give diagnostic criteria, if applicable | 4,5 | This has been indicated under the sections Data Collection and Blood testing procedures. |
| Data sources/ measurement | 8* | For each variable of interest, give sources of data and details of methods of assessment (measurement). Describe comparability of assessment methods if there is more than one group | *5* | This has been indicated under the sections Blood testing procedures, and Data Collections. |
| Bias | 9 | Describe any efforts to address potential sources of bias | 5,6 | This has been indicated under the sections Data Collections and under data management and analysis section. |
| Study size | 10 | Explain how the study size was arrived at | 4 | This has been indicated under the section survey population |

Continued on next page

| Quantitative variables | 11 | Explain how quantitative variables were handled in the analyses. If applicable, describe which groupings were chosen and why  This has been indicated under the sections under the data management and analysis in page 6. |  |  |
| --- | --- | --- | --- | --- |
| Statistical methods | 12 | 1. Describe all statistical methods, including those used to control for confounding   This has been indicated under the sections under the data management and analysis in page 6. |  |  |
|  |  | 1. Describe any methods used to examine subgroups and interactions   This has been indicated under the sections under the data management and analysis on page 6. |  |  |
|  |  | 1. Explain how missing data were addressed   This has been indicated under the sections under the data management and analysis in page 6. |  |  |
|  |  | (*d*) *Cohort study*—If applicable, explain how loss to follow-up was addressed  *Case-control study*—If applicable, explain how matching of cases and controls was addressed  *Cross-sectional study*—If applicable, describe analytical methods taking account of sampling strategy  This has been indicated under the sections under the data management and analysis in page 6. |  |  |
|  |  | 1. Describe any sensitivity analyses   There was no sensitivity analysis done. |  |  |
| Results | | | | |
| Participants | 13* | 1. Report numbers of individuals at each stage of study—eg numbers potentially eligible, examined for eligibility, confirmed eligible, included in the study, completing follow-up, and analysed   This has been indicated under the sections under the demographic characteristics in page 7 |  |  |
|  |  | 1. Give reasons for non-participation at each stage   This has been indicated under the sections under the demographic characteristics in page 7 |  |  |
|  |  | 1. Consider use of a flow diagram   Diagram was not necessary. |  |  |
| Descriptive data | 14* | 1. Give characteristics of study participants (eg demographic, clinical, social) and information on exposures and potential confounders   This has been indicated under the sections under the demographic characteristics in page 7 and 8 |  |  |
|  |  | 1. Indicate number of participants with missing data for each variable of interest   N/A |  |  |
|  |  | (c) *Cohort study*—Summarise follow-up time (eg, average and total amount) |  |  |
| Outcome data | 15* | *Cohort study*—Report numbers of outcome events or summary measures over time |  |  |
|  |  | *Case-control study—*Report numbers in each exposure category, or summary measures of exposure |  |  |
|  |  | *Cross-sectional study—*Report numbers of outcome events or summary measures  This has been indicated in page 7- 11 |  |  |
| Main results | 16 | 1. Give unadjusted estimates and, if applicable, confounder-adjusted estimates and their precision (eg, 95% confidence interval). Make clear which confounders were adjusted for and why they were included   This has been indicated in page 10- 11 |  |  |
|  |  | 1. Report category boundaries when continuous variables were categorized   This has been indicated in page 10- 11 |  |  |
|  |  | (*c*) If relevant, consider translating estimates of relative risk into absolute risk for a meaningful time period |  |  |

Continued on next page

| Other analyses | 17 | Report other analyses done—eg analyses of subgroups and interactions, and sensitivity analyses  N/A |  |  |
| --- | --- | --- | --- | --- |
| Discussion | | | | |
| Key results | 18 | Summarise key results with reference to study objectives  This has been indicated in page 12 |  |  |
| Limitations | 19 | Discuss limitations of the study, taking into account sources of potential bias or imprecision. Discuss both direction and magnitude of any potential bias  This has been indicated in page 13 at the end of the discussion |  |  |
| Interpretation | 20 | Give a cautious overall interpretation of results considering objectives, limitations, multiplicity of analyses, results from similar studies, and other relevant evidence  This has been indicated in page 12-14 |  |  |
| Generalisability | 21 | Discuss the generalisability (external validity) of the study results  This has been indicated in page 13 |  |  |
| Other information | |  | | |
| Funding | 22 | Give the source of funding and the role of the funders for the present study and, if applicable, for the original study on which the present article is based  This has been indicated in the additional information during the submission |  |  |

*Give information separately for cases and controls in case-control studies and, if applicable, for exposed and unexposed groups in cohort and cross-sectional studies.

**Note:** An Explanation and Elaboration article discusses each checklist item and gives methodological background and published examples of transparent reporting. The STROBE checklist is best used in conjunction with this article (freely available on the Web sites of PLoS Medicine at http://www.plosmedicine.org/, Annals of Internal Medicine at http://www.annals.org/, and Epidemiology at http://www.epidem.com/). Information on the STROBE Initiative is available at www.strobe-statement.org.
